# Exploring the relationship between cultural landscapes and psychological well-being through indigenous environmental design in rural China

**DOI:** 10.3389/fpsyg.2026.1978816

**Published:** 2026-10-29

**Authors:** Xinyao Wang, Yudan Zhu

**Affiliations:** 1 School of Culture Tourism and Journalism Arts (Humanities Education Center), Shanxi University of Finance and Economics, Taiyuan, China; 2 Architecture and Environmental Arts, Shanghai Urban Construction Vocational College, Shanghai, China

**Keywords:** indigenous environmental design, cultural landscapes, psychological well-being, place attachment, perceived restorativeness, cultural identity continuity, ritual-spatial synchrony, social cohesion

## Abstract

**Introduction:**

Indigenous environmental design in rural China is increasingly affected by modernization, tourism development, and ecological change. However, empirical evidence concerning the relationship between cultural landscape fidelity and residents’ psychological well-being remains limited. This study developed the Cultural Landscape–Psychological Well-being (CL-PWB) Nexus Model to examine the association between Indigenous Environmental Design Fidelity (IEDF) and psychological well-being, considering place attachment, perceived restorativeness, and cultural identity continuity as mediators, and ritual-spatial synchrony (RSS) and social cohesion (SC) as potential moderators.

**Methods:**

A sequential explanatory mixed-methods design was employed. The quantitative phase involved a cross-sectional survey of 420 residents from four ethnic minority villages in rural China. Confirmatory factor analysis and regression-based moderated mediation analysis with bootstrapped confidence intervals were used to examine the proposed relationships. The qualitative phase comprised thematic analysis of 40 in-depth and key-informant interviews to contextualise and complement the quantitative findings.

**Results:**

IEDF was positively associated with psychological well-being. This association was statistically accounted for by place attachment, perceived restorativeness, and cultural identity continuity, with the direct association becoming non-significant after the mediators were included. Conditional indirect associations were generally stronger at higher levels of RSS and SC, although the patterns varied across the mediating pathways. At lower levels of RSS and SC, the indirect association through place attachment was no longer statistically distinguishable from zero, whereas the associations through perceived restorativeness and cultural identity continuity remained significant but weaker. Four qualitative themes—ancestral memory, designed restoration, ritual erosion, and communal cohesion—provided contextual support for the quantitative finding.

**Discussion:**

The findings suggest that the relationship between indigenous environmental design and psychological well-being extends beyond the preservation of physical landscape features to include place-based emotional connections, restorative experiences, cultural continuity, ritual practices, and community interaction. Given the cross-sectional and self-reported nature of the data, the findings indicate associations rather than causal effects. The study provides an empirical basis for longitudinal and multilevel investigations and suggests that culturally informed rural development and mental-health policies should consider both the material and lived dimensions of indigenous cultural landscapes.

## 1 Introduction

Throughout the world, ethnic-minority communities have long structured their built and natural environments in ways that go beyond their material needs for shelter and subsistence based on cosmological, ecological, and social principles. Ethnic minority groups like the Dong, Miao, Buyi and Naxi have maintained unique design traditions for their environment, including fengshui forests, drum towers, ‘wind and rain bridges’, terracing of agricultural fields and settlement patterns based on ancestral and spiritual considerations, which represent a wealth of knowledge passed down through generations about how people need to live in relation to the land, their community and the sacred. These scenery are living infrastructures of meaning, which shape everyday life, seasonal ritual and passing cultural identity from generation to generation (Gu et al., 2025). As rural China rapidly modernizes its infrastructure, changes in its ecosystems, and continues to be reshaped by tourism, however, many of these cultural landscapes are being transformed, fragmented, or abandoned (Ma et al., 2023), and the disruption of rural life caused by these changes has been shown to have measurable impacts on mental wellbeing among residents (Deng et al., 2023; Zhou et al., 2022). When these design traditions are lost, what is lost too — not only culturally, but psychologically?

Modern psychological researchers have increasingly realised that mental health and well-being are not solely related to an individual’s thoughts or clinical symptoms, but are influenced by a complex of social and environmental factors, and culture and spiritual values, which have historically not been the focus of mainstream psychology. From refugee children who are coping with resettlement (Albers et al., 2021), to older adults who are ageing in place (Chen et al., 2024), to migrant children who are experiencing discrimination (Yang et al., 2023), place attachment has been consistently found to have a protective and/or positive impact on well-being for a wide range of populations. Environmental restorativeness has also been shown to have associations with psychological recovery from attentional and emotional fatigue (Johnson et al., 2022), and cultural continuity has emerged as a strong protective factor for mental health disparities among Indigenous communities specifically (Firestone et al., 2022; Hodgson et al., 2022). However, these three mechanisms are not often considered in concert, nor is ethnic-minority environmental design in the specific context of an intentional, designed, and constructed system, and not as a backdrop. Furthermore, there has been a tendency in existing well-being studies in Indigenous contexts to focus on strengths-based cultural interventions (e.g., Rieger et al., 2023; Summerton and Blunden, 2022) and on community-level service delivery models (e.g., O’Keefe et al., 2021) without properly theorizing how the physical fidelity of a cultural landscape relates to the extent to which that landscape is being actively used and is being communally sustained. This disconnection restricts the field’s ability to speak to questions of meaningfulness and connection in relation to the spaces that remain in good physical condition while others are more psychologically connected to their traditional spaces.

This gap is the main issue the present study seeks to explore. While there is an increasing theoretical consensus that culture and environment cannot be separated from mental health, there is still a great deal of disconnect between this acceptance and the understanding of how certain aspects of Indigenous environmental design relate to measurable psychological outcomes, and under what conditions they translate successfully and under what conditions they do not. Despite increasing recognition of the importance of traditional ecological knowledge as resources for psychological well-being in communities (Carroll et al., 2025), there is little research on how the design features through which traditional ecological knowledge is embodied in the landscape relate to residents’ psychological well-being. A recent mixed-methods study of 180 Miao, Dong, and Naxi participants in Southwest China reported that heritage-tourism participation was positively correlated with psychological well-being, resilience, perceived social support, and cultural identity, and its qualitative themes included traditional healing, social cohesion and belongingness, and ecological attachment (Zhao et al., 2026). That study took heritage-tourism participation, rather than the fidelity of the landscape itself, as its focal exposure, and relied on correlation and regression analyses rather than a mediation–moderation model. Without such understanding, health and well-being programmes may inadvertently focus on preserving cultural landscapes as heritage objects rather than as dynamic social and psychosocial resources whose value may depend on ongoing ritual use and social cohesion; existing studies suggest that social cohesion and social capital are related to well-being in different ways in rural China, depending on location and demographic characteristics (Burnette et al., 2021; Xia et al., 2024; Xu et al., 2023). These communities in the rural areas of China that are facing modernization pressures provide a very relevant context for the research of this issue, not only because they are rich in the practices of environmental design that still exist but also because they are rapidly changing as infrastructural and demographic modernization are reshaping them. The present study therefore aims to specify conditions under which well-established pathways (place attachment, perceived restorativeness, and cultural continuity) are associated with well-being in relation to landscape fidelity, rather than to propose entirely new mechanisms.

Based on this, the following key research question is set: What is the relationship between Indigenous environmental design in the rural communities in China and the psychological status of their residents, and how and under which conditions does this relationship exist? This question is then broken down into three sub-questions: (1) To what extent does the fidelity of Indigenous environmental design predict psychological well-being both directly and indirectly; (2) How does this relationship play out through psychological mechanisms such as Place Attachment, Perceived Restorativeness, and Cultural Identity Continuity; and (3) What conditions of ritual-spatial practice and social cohesion strengthen or weaken this relationship?

Hence, the main objectives of this study are as follows: The first of these is to create and empirically test a novel conceptual model, the Cultural Landscape – Psychological Well-being (CL-PWB) Nexus Model, which identifies indigenous environmental design fidelity as a predictor of psychological well-being, mediated by three concurrent mechanisms. The second is to consider ritual-spatial synchrony and social cohesion as boundary conditions that moderate the mediation between the two, which has been tested in both primary and secondary mediation. The third is to supplement this quantitative structural model with a detailed qualitative study (storytelling, interviews) of the experiences of residents in terms of how cultural landscapes affect their psychological experience, to anchor statistical pathways in lived meaning, with a focus on lived meaning that is consistent with best practice in Indigenous health research (Rieger et al., 2023). A fourth and final goal is to bring together these quantitative and qualitative findings to produce practical knowledge for culturally inclusive mental health and rural heritage policy and contribute to the larger goal of treating these communities’ design knowledge as a valuable and neglected resource for the contemporary mental health conversation.

*Terminology*: In this article, ‘Indigenous environmental design’ refers to long-established, place-based design traditions of ethnic minority communities in Southwest China (for example, drum towers, wind-and-rain bridges, fengshui forests, and terraced settlements). In China the official category is ‘ethnic minority’ (shaoshu minzu); following the Indigenous psychology and Indigenous health literature cited here, ‘Indigenous’ is used as an analytic label for these design traditions, not as a claim that the Dong, Miao, Buyi, and Naxi share a single cultural framework or that the term carries the same political or legal meaning as in settler-colonial contexts (Smith, 2012). ‘Indigenous Environmental Design Fidelity’ (IEDF) denotes the extent to which a village’s built and natural environment retains the spatial cosmology, material authenticity, and ritual-functional integration of its design tradition. It is measured here by residents’ ratings (perceived fidelity; it is distinct from formal preservation or heritage-designation status and from ritual use, which is captured separately by ritual-spatial synchrony (RSS). Whether the constructs carry equivalent meaning across the four groups is an empirical question that this study can address only partly (see Limitations). Indigenous Environmental Design Fidelity (IEDF) was operationalised as a resident-reported construct reflecting the perceived preservation of spatial cosmology, material authenticity, and ritual-functional integration within the village landscape. The construct was measured using a structured five-point Likert scale, with higher scores indicating greater perceived fidelity. This measure captures residents’ subjective experience of the cultural landscape rather than an independent architectural or heritage-conservation assessment.

## 2 Literature review

### 2.1 Place attachment as a well-being mechanism

There is an empirical trend of place attachment being protective and enhancing for highly diverse populations. Based on two-wave SEM results for older adults in Taiwan, Chen et al. (2024) concluded that the link between attitudes toward ageing and subjective well-being was mediated by place attachment. Likewise, in a two-wave longitudinal study, Yang et al. (2023) showed that place attachment (place dependence) was a protective factor against psychological distress resulting from perceived discrimination for Chinese migrant children. These findings are also echoed in the context of heritage sites: Jin et al. (2025) showed that living-environment quality of traditional Hangzhou villages indirectly explained the cultural-heritage responsibility behaviours of residents through the mediation of place attachment and pride, and Cai et al. (2025) demonstrated that place attachment mediated the relationship between living-environment quality and sustainable behaviours toward marine cultural heritage in a fishing village in Fujian. Although there is a consistent structural logic in the studies comparing place attachment as a mediator rather than as a direct predictor, there is a gap: none of the studies compares the strength of attachment of individuals who merely live in or have been designated as a “heritage site” with individuals who experience the site as faithful according to the IEDF construct introduced in this paper.

### 2.2 Restorative and nature-connectedness pathways

A second set of studies builds on Attention Restoration Theory and on nature-connectedness research in Chinese populations. These studies concern nature connectedness rather than the perceived restorativeness of culturally designed settings, and are cited for the broader logic linking nature-related experience to well-being. Zhu et al. (2025) tested a moderated mediation model in 401 older adults in China, in which connectedness to nature was positively associated with psychological well-being, with self-perceptions of aging as a mediator and meaning in life as a moderator. Yang et al. (2022) studied 675 rural low-income residents in Jiangsu and found that affect balance and neighbourhood social cohesion sequentially mediated the association between connectedness to nature and life satisfaction or depression, although the serial mediation effect was small. Yuan et al. (2026) examined Yi ethnic minority pupils in rural Southwest China and found that empathy mediated the association between nature connectedness and social–emotional competence, which highlights nature-based mechanisms in an ethnic minority context, but one focused on children’s social–emotional competence and not on adult well-being. The adult, place-based restorativeness question addressed here therefore remains largely unexamined in ethnic minority settings.

### 2.3 Cultural landscapes and traditional villages in rural China

The literature available is under-integrated and focuses on the ethnic minority villages of China mostly from planning, heritage-conservation and sustainability perspectives instead of psychological perspectives. Gu et al. (2025) were able to map out the spatial organization of “cultural landscape genes” in Goulan Yao Village, uncovering a “three-core, multi-node” spatial pattern that requires a conservation strategy between symbolic preservation and spatial accessibility. Ma et al. (2023) provide evidence of erosion in Hani terraced villages under urbanisation pressure, through quantification of the decrease in ecosystem-service value and increase in landscape fragmentation. In either study, however, psychological outcomes have not been measured; they simply describe what is being lost from cultural landscapes, but without consideration of what that loss means to residents’ psychological well-being, which is precisely the focus of the present mixed-methods design and the effect of the link between IEDF (a fidelity construct that is compatible with a landscape-gene perspective) and measured outcomes.

### 2.4 Ethnic minority and indigenous cultural identity

There is a strand of empirical research on the psychological function of cultural identity for ethnic minorities in China and for Indigenous groups elsewhere. Luo et al. (2024) studied Yi minority youth in China (a group not sampled in the present study) and found that intergroup orientation mediated the association between ethnic and national identification, indicating that identity has psychological consequences and not only demographic ones. Gonzalez et al. (2022) proposed ‘cultural efficacy’ as a construct linking enculturation with lower anxiety and better mental health in Indigenous communities in the upper Midwest and Canada, which is conceptually close to the cultural identity continuity mediator used here. In Southwest China, Zhao et al. (2026) studied 180 Miao, Dong, and Naxi participants (groups that overlap with those sampled here) and reported positive correlations between heritage-tourism participation and psychological well-being (*r* = 0.38), resilience (*r* = 0.31), perceived social support (*r* = 0.28), and cultural identity (*r* = 0.34); their qualitative themes also included social cohesion and belongingness, traditional healing, and ecological attachment. Among the Naga, Yepthomi et al. (2025) described healing modalities, such as communal and cosmological systems, that lie outside the biomedical model. Read side by side, these studies agree that culture is associated with protection, but they differ on what the active ingredient may be: efficacy or agency in Gonzalez et al. (2022), and tourism participation in Zhao et al. (2026). The present study asks whether the fidelity of the landscape and the spatial persistence of ritual practice may be among the conditions that such constructs reflect; this question is empirical, and our cross-sectional data can address it only through associations.

### 2.5 Social cohesion and rural community well-being in China

A final cluster confirms the relevance of well-being for social cohesion in the context of the rural population in China, in keeping with the moderator used in this research. Based on the CGSS data from all of China, Xu et al. (2023) determined that social capital was an intervening variable between trust and happiness among rural residents. Xia et al. (2024) determined that socioeconomic security, cohesion, inclusion and empowerment were not equally important predictors of life satisfaction among rural elderly. Burnette et al. (2021) found that social cohesion mediated the association between living arrangement and quality of life among older adults in urban but not rural China, which suggests that the role of social cohesion may differ by context; the present study examines cohesion as a moderator within an exclusively rural sample. This line was further extended by Wan et al. (2025) to community elderly-care services and by Sun et al. (2026) to social alienation, with both studies finding psychological resilience/subjective well-being as a strong mediator in rural Chinese populations. Deng et al. (2023) and Zhou et al. (2022) bring a migration lens, revealing that for rural people, experiencing migration has a negative impact on their mental health, whereas for new-generation migrants, social integration has a positive effect, suggesting that social ties and integration matter for the well-being of both rural and migrant populations.

### 2.6 Synthesis and gap

Organised around the central questions of this study, the literature converges and diverges as follows. On the question of which place-based mechanisms are associated with well-being, studies of place attachment and nature connectedness agree that attachment to, and connection with, places is positively associated with well-being across diverse populations in China and in Indigenous communities elsewhere (Chen et al., 2024; Yang et al., 2022, 2023; Zhu et al., 2025), and studies of cultural identity and enculturation point in the same direction (Gonzalez et al., 2022; Zhao et al., 2026). On the question of the conditions under which these associations hold, findings diverge: the role of social cohesion appears to depend on context, such as urban versus rural settings and migrant versus resident status (Burnette et al., 2021; Xia et al., 2024; Xu et al., 2023), and landscape-erosion studies show what is being lost without measuring its psychological consequences (Gu et al., 2025; Ma et al., 2023). What remains unresolved is whether, and under what conditions, the fidelity of a designed cultural landscape and the persistence of ritual practice within it (RSS) are jointly related to well-being through these mechanisms. The studies closest to our setting, the heritage-tourism study of Zhao et al. (2026) and the Yi-pupil study of Yuan et al. (2026), document well-being correlates without testing a mediated, conditional model of landscape fidelity. This is the gap addressed by the CL-PWB Nexus Model.

### 2.7 Hypothesis development

Three theoretical frameworks are built as the foundation for this study: Place Attachment Theory, Attention Restoration Theory (ART), and Cultural Continuity Theory, along with some recent empirical studies with a proven ability to extract insights from the Chinese traditional village field.

Place Attachment Theory (Williams and Vaske, 2003; Scannell and Gifford, 2010; see also Tuan, 1977; Hidalgo and Hernández, 2001; Lewicka, 2011) suggests that the emotional relationship between a person and a meaningful place (place identity and place dependence) is a powerful determinant of a person’s psychological functioning and self-continuity. Twigger-Ross and Uzzell (1996) also demonstrated that the feelings of continuity, self-esteem and distinctiveness associated with attachment to residential and/or ancestral places helped strengthen identity principles and that when these attachments are disrupted, a grief-like reaction occurs. In the context of Indigenous environmental design, the following recent studies of traditional and ethnic minority villages of China are consistent with this logic: Wang et al. (2025) discovered that, in the case of the Dong village in Guizhou, farmers’ sense of place is enacted around the drum tower and altar, which are among the traditional architectures; Xu et al. (2021) and Wang et al. (2019) documented that disruption of the traditional rural landscape through resettlement measurably weakens farmers’ sense of place; and Zhu et al. (2021) also demonstrated the same effects among the resettled communities in Guizhou Province in particular. Based on the known link between place attachment and well-being, we hypothesize that:

*H1*: There is a positive relationship between Indigenous Environmental Design Fidelity (IEDF) and psychological wellness.

*H2a*: There is a positive relationship between IEDF and place attachment.

*H4a*: A positive relationship exists between place attachment and psychological well-being.

To reduce stress and replenish depleted attention resources, certain environments are proposed as being capable of restoring attention, based on Attention Restoration Theory (Kaplan, 1995; Kaplan and Kaplan, 1989; for a review see Hartig et al., 2014), which includes the features of being-away, fascination, extent, and compatibility. The study by Ulrich (1984) was the first to provide early empirical evidence for the restorative environment effect, since it showed that passive visual contact with a natural setting improved patients’ outcomes of recovery. Although ART focuses mainly on natural environments, culturally designed environments has plausibly have similar restoration effects if they are experienced as a coherent, and psychologically remote from daily demands, environment, which is consistent with Zhou et al. (2023), who reported positive emotional perceptions of space atmosphere among visitors to traditional Chinese settlements, as well as Lin (2024), who examined how the space atmosphere of traditional villages and tourists’ attachment to place relate to revisit intention. Although ART focuses mainly on natural environments, culturally designed environments may have similar restorative effects when they are experienced as coherent and psychologically removed from everyday demands. This reading is consistent with Zhou et al. (2023), who reported positive emotional perceptions of spatial atmosphere among visitors to traditional Chinese settlements, and with Lin (2024), who found that spatial atmosphere and place attachment were associated with tourists’ well-being and revisit intention in traditional villages; that study, however, examined visitors rather than residents, and its outcome was revisit intention. This suggests:

*H2b*: IEDF is positively correlated with perceived restorativeness.

*H4b*: Psychological well-being is positively related to perceived restorativeness.

Cultural Continuity Theory (Chandler and Lalonde, 1998) suggests that psychological resilience is very much tied to the capacity of a community to maintain continuity between past, present and future, and to control cultural, linguistic and territorial markers of identity. This directly encourages cultural identity continuity as another, more collective mediator, separate from the individually oriented mediators mentioned above. These collective dimensions are confirmed by recent case studies in China: Xiang et al. (2022) reported direct links between public ritual spaces and social cohesion in Dong villages and Xu et al. (2023) observed that tourists’ and residents’ spiritual connections to traditional villages are built by engaging in a continuum of situational and ritual immersion, rather than passive immersion. Accordingly:

*H2c*: IEDF is positively related to cultural identity continuity.

*H4c*: There is a positive relationship between maintaining cultural identity continuity and psychological well-being.

These three pathways suggest a general mediation hypothesis:

*H6*: Place attachment, perceived restorativeness, and cultural identity continuity mediate the relationship between IEDF and psychological well-being.

In addition to these direct and mediating relationships, this study suggests that the effects of the pathways are not determined but are also conditional. Chandler and Lalonde (1998) identify two types of continuity: symbolic continuity (structures remain physically unchanged) and behavioural continuity (structures continue to be used in ritual ways). The latter form of continuity has been suggested as the true protective factor, an empirical finding supported by Jin et al. (2025), who found that living-environment quality was predictive of cultural-heritage responsibility behavior through active place attachment and pride, not directly; and Lu et al. (2024) who found that the mediating effect of local identity (actively felt but not directly expressed) between living-environment quality and cultural heritage conservation behavior was more significant than the mediating effect of landscape presence. Based on this difference, we propose a first-stage moderator, ritual spatial synchrony (RSS):

*H3*: The relationship between IEDF and each mediator is moderated by ritual-spatial synchrony such that the relation is stronger if practices are still spatially linked to traditional practices.

Likewise, Sampson et al. (1997) found that collective efficacy (social cohesion and shared disposition to act) influences community-level outcomes, over and above individual characteristics; and Kawachi and Berkman (2001) found that social ties buffer and amplify the mental-health advantages of supportive environments. Similarly, Shi’s (2025) examination of tourist sense-of-place in ecologically designed villages revealed that value and identity effects on attitudes were moderated by a sense of communal involvement with the site rather than the physical presence of the site. This encourages another stage moderator, social cohesion (SC):

*H5*: Social cohesion plays a moderating role between each mediator and psychological well-being, such that the relationship between each mediator and psychological well-being is stronger when social cohesion is high.

First and second stage moderation hypotheses combine to lead to the study’s integrative prediction:

*H7*: The indirect effect of IEDF on psychological well-being through each of the three mediators (place attachment, perceived restorativeness, and cultural identity continuity) is conditional on both moderators: it is stronger at higher levels of ritual-spatial synchrony (first stage) and at higher levels of social cohesion (second stage), as indicated by the index of moderated mediation for each stage.

The outcome construct is grounded in Ryff's (1989) six-dimensional model of psychological well-being (autonomy, environmental mastery, purpose in life, positive relations, self-acceptance, and personal growth) in relation to all seven hypotheses (see Figure 1).

**Figure 1:**
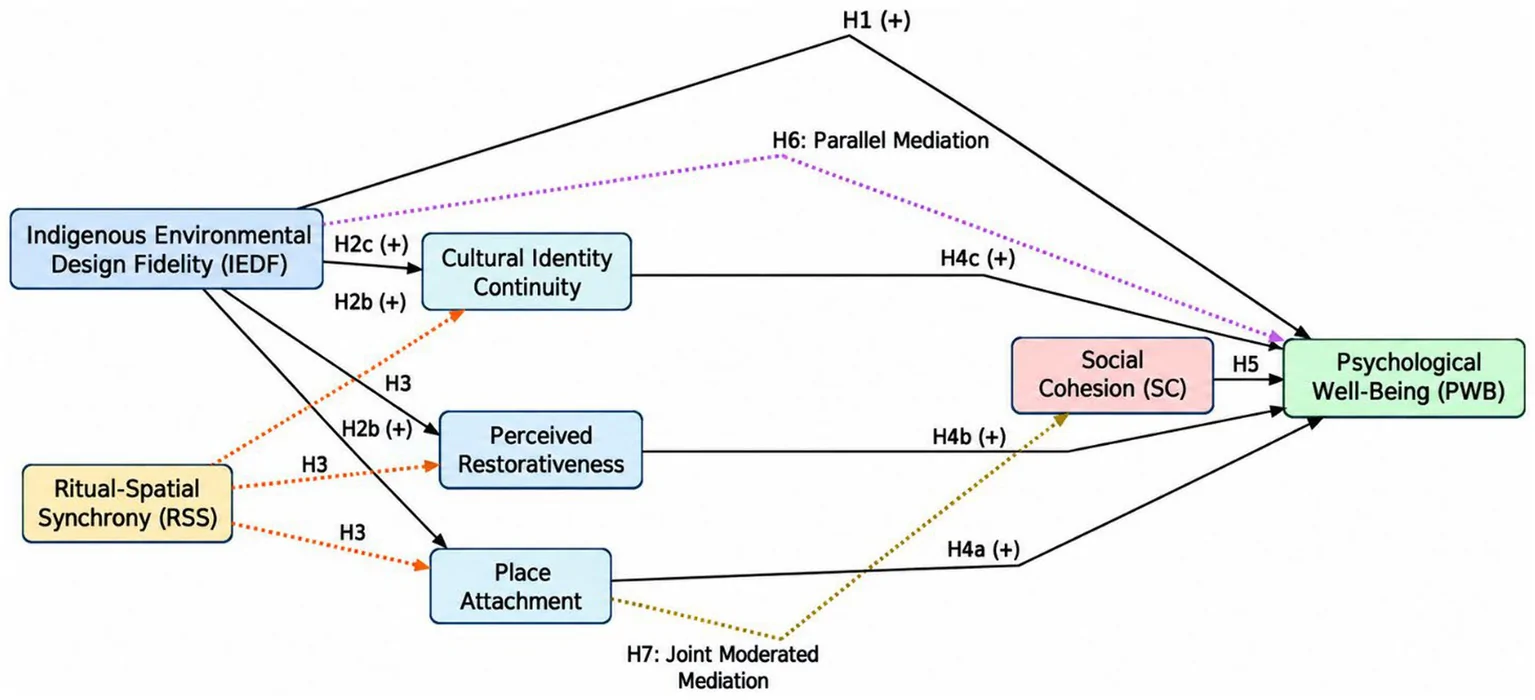
Conceptual framework diagram.

## 3 Research methodology

The study used a mixed-methods sequential explanatory design (QUAN → qual): a cross-sectional survey, analysed with confirmatory factor analysis and regression-based moderated-mediation (path) analysis, was followed by semi-structured interviews with residents and key informants. This sequence allowed the study to test a conceptual model statistically and then use interpretive, narrative accounts (ritual, storytelling, memory) to help explain the quantitative patterns, in line with approaches central to Indigenous psychology. Fieldwork was conducted in four villages in rural Southwest China (designated Villages A–D here to protect community anonymity). Villages were selected for their recognised ethnic minority populations (Dong, Miao, Buyi, or Naxi) and for retaining recognisable features of Indigenous environmental design, including fengshui forests, drum towers, wind-and-rain bridges, terraced agriculture, and cosmologically organised settlement patterns. Sites were chosen through a maximum-variation strategy on three dimensions (extent of preservation of traditional environmental design, exposure to rural tourism, and ethnic-cultural distinctiveness) so that outcomes could be compared across different levels of cultural-landscape preservation. Because the villages differ simultaneously in culture, tourism, and environment, these village-level differences cannot be separated from one another in this design (see Limitations).

A multistage stratified purposive sampling method is used. Villages are selected based on the above criteria in a purposive manner at Stage 1. To ensure variation in everyday exposure to the restorative environment and ritual cues, at Stage 2 households were stratified according to GIS-measured proximity to the core cultural-landscape feature (within 200 m, 200–500 m, or beyond 500 m). The proximity strata were used for sampling only; proximity was not analysed as a variable in this article. A Kish grid is used at Stage 3 to ensure there is minimal self-selection bias, with one eligible resident (aged 18 years and older with 5 years or more of continuous residence) selected per household. The sample size was determined *a priori* using G*Power 3.1 for the fullest conditional regression model (including interaction terms) assuming a medium effect size (f^2^ = 0.15), α = 0.05, and power = 0.95, which results in a minimum sample size of 153 cases; this was expanded to meet the sample-to-parameter ratio rule (10 cases per free parameter) for the full structural model (c. 34 parameters, *n* ≥ 340), and then increased by 20% to allow for missing data and rural-field-work attrition. The final quantitative sample consists of *n* = 420 respondents, divided across the four villages of the study, with about 35 respondents per proximity tertile in each village. The qualitative subsample consisted of a fixed sample of 32 participants (8 per village: 4 elders and 4 younger adults, who were also classified by survey scores as high (*n* = 4) or low (*n* = 4) in place attachment, so that the age and place-attachment categories overlap) and 8 expert key informants (ritual specialists, elder carpenters, and heritage officers) who were recruited through snowball sampling and checked on a running thematic-saturation grid, to reach a total of 40 participants.

*Sample composition*: The analysed sample comprised 420 respondents, 105 in each of the four villages (A, Dong; B, Miao; C, Buyi; D, Naxi) and 35 in each proximity stratum within each village; 204 were women and 216 were men. Mean age was 44.3 years (SD = 13.65; range 18–88), mean length of residence was 35.0 years (SD = 14.51), and 35% reported migration exposure. The study was conducted in four ethnic minority villages in rural China, identified in the manuscript as Villages A, B, C, and D. These locations represented Dong, Miao, Buyi, and Naxi cultural contexts, respectively. The villages were selected to examine the relationship between culturally embedded environmental design, ritual-spatial practices, social cohesion, and residents’ psychological well-being. The quantitative survey and qualitative interviews were conducted during August to November 2025. Village identifiers were anonymized in the manuscript to maintain confidentiality and protect community-level information. The quantitative phase included 420 residents from four ethnic minority villages, with 105 respondents recruited from each village. The sample comprised 204 women and 216 men. Participants had a mean age of 44.30 years (SD = 13.65), with ages ranging from 18 to 88 years. The mean duration of residence in the respective villages was 34.96 years (SD = 14.51). Approximately 35% of respondents reported migration-related exposure. The distribution across the four villages provided equal numerical representation for the comparative study design. The sample was used to examine the associations among Indigenous Environmental Design Fidelity, ritual-spatial synchrony, social cohesion, the proposed mediating variables, and psychological well-being.

The quantitative instrument battery consists of Ryff’s Psychological Well-Being Scales (Chinese-validated short form), the Place Attachment Scale adapted from Williams and Vaske, an adapted Perceived Restorativeness Scale, an original Indigenous Environmental Design Fidelity Index (2-round Delphi process with key informants), a Ritual-Spatial Synchrony checklist documenting the frequency and locational fidelity of named practices, and a Social Cohesion scale completed by each respondent about their own village (analysed here as an individual-level perception of cohesion). All instruments are sent for forward-backwards translation between Mandarin and relevant minority languages and cognitive interviewing before a pilot of 40 participants, in which Cronbach’s α ≥ 0.80 was set as the target for retaining a scale (Boateng et al., 2018). A pilot assessment involving 40 participants was undertaken to evaluate the clarity, comprehensibility, and preliminary reliability of the measurement instruments. The pilot process was intended to identify ambiguous items, assess the suitability of the response format, and determine whether the measures were appropriate for the cultural and linguistic context of the study. The internal consistency target was set at Cronbach’s alpha ≥ 0.80. The pilot findings were considered during instrument refinement before the main survey.

A major component of the methodology is the Cultural Landscape – Psychological Well-being Nexus Model (CL-PWB Nexus Model) that considers indigenous environmental design as a psychosocial mechanism that is active rather than passive, mediated and moderated. The exogenous predictor is Indigenous Environmental Design Fidelity (IEDF), which is a composite that reflects spatial cosmology, material authenticity, and ritual-functional integration. The total association of IEDF with psychological well-being (PWB) is estimated as

$$\mathrm{PWB}=\beta_{0}+c\cdot \text{IEDF}+\beta \_\mathrm{CV}\cdot \mathrm{CV}+\varepsilon ₁$$

where CV denotes covariates (age, gender, log-transformed income, and migration exposure). Three parallel mediators — place attachment (PA), perceived restorativeness (PR), and cultural identity continuity (CIC) — are each regressed on IEDF via the a-path equation

$$M_{i}=\beta_{0i}+a_{i}\cdot \text{IEDF}+\beta \_CV_{i}\cdot \mathrm{CV}+\varepsilon_{i}\;$$

PWB is then regressed on IEDF together with all three mediators via the b-path equation

$$\mathrm{PWB}=\beta_{0}+c^{\prime }\cdot \text{IEDF}+b₁\cdot \mathrm{PA}+b₂\cdot \mathrm{PR}+b₃\cdot \mathrm{CIC}+\beta \_\mathrm{CV}\cdot \mathrm{CV}+\varepsilon ₂$$

yielding the simple indirect effect for each mediator as $\text{Indirec}t_{i}=a_{i}\cdot b_{i}$ and a total indirect effect of $\Sigma (a_{i}\cdot b_{i})$ .

Two elaborations of this simple mediation pattern, both motivated by theory, are presented. In this study, a new moderator construct, called Ritual-Spatial Synchrony (RSS), was created, which models whether daily and ceremonial activities are still carried out at historically designated landscape locations, and was used as a first-stage moderator of the IEDF–mediator paths:

$$\begin{array}{l} M_{i}=\beta_{0i}+a_{1i}\cdot \text{IEDF}+a_{2i}\cdot \mathrm{RSS}+a_{3i}\cdot (\text{IEDF}\times \mathrm{RSS})\\ +\beta \_CV_{i}\cdot \mathrm{CV}+\varepsilon_{i} \end{array}$$

so that the conditional a-path becomes $a_{i}(\mathrm{RSS})=a_{1i}+a_{3i}\cdot \mathrm{RSS}$ . Social Cohesion (SC) is modelled as a second-stage moderator of the mediator–PWB paths:

$$\begin{array}{l} \mathrm{PWB}=\beta_{0}+c^{\prime }\cdot \text{IEDF}+b_{1i}\cdot M_{i}+b_{2i}\cdot \mathrm{SC}\\ +b_{3i}\cdot (M_{i}\times \mathrm{SC})+\beta \_\mathrm{CV}\cdot \mathrm{CV}+\varepsilon ₂ \end{array}$$

giving a conditional b-path of $b_{i}(\mathrm{SC})=b_{1i}+b_{3i}\cdot \mathrm{SC}$ . Combining both stages produces the conditional indirect effect $\text{Indirec}t_{i}(\mathrm{RSS},\mathrm{SC})=[a_{1i}+a_{3i}\cdot \mathrm{RSS}]\times [b_{1i}+b_{3i}\cdot \mathrm{SC}]$ , and the index of moderated mediation is calculated separately for each stage as $\omega_{1i}=a_{3i}\cdot b₁$ for first-stage moderation and $\omega_{2i}=a_{1i}\cdot b₃$ for second-stage moderation; a significant index, evidenced by a 95% bias-corrected bootstrap confidence interval excluding zero, indicates that the conditional indirect association differs across levels of RSS or SC, rather than relying on arbitrary probing values.

*Analytic approach*: The seven-factor measurement model was evaluated with confirmatory factor analysis (CFA) using maximum likelihood estimation with robust standard errors (MLR), with model fit assessed against CFI/TLI > 0.95, RMSEA < 0.06, and SRMR < 0.08. The structural hypotheses were then tested with the regression-based path-analytic (moderated-mediation) system specified above, estimated by ordinary least squares on observed composite scores (Edwards and Lambert, 2007; Hayes, 2018; Preacher et al., 2007). The quantitative analysis was conducted in two stages in SPSS and AMOS. First, descriptive statistics, correlation analysis, and confirmatory factor analysis were used to examine the distributions, relationships, and measurement properties of the study constructs. The measurement model was assessed using standardized factor loadings, internal consistency, composite reliability, and average variance extracted.

Second, regression-based path analysis was used to examine the proposed direct, indirect, and conditional associations. The indirect effects were evaluated using 5,000 bootstrap resamples and bias-corrected 95% confidence intervals. The moderation analyses examined the conditional associations of ritual-spatial synchrony and social cohesion with the proposed pathways. Continuous predictors were mean-centered or standardized according to the analytical procedure described in the manuscript.

RSS and SC were standardized, so that the ‘mean’ condition corresponds to 0 and the ‘low’ and ‘high’ conditions to −1 SD and +1 SD; conditional indirect effects were evaluated with RSS and SC set to the same level. Indirect, conditional indirect, and index-of-moderated-mediation effects were estimated with 5,000 bootstrap resamples and bias-corrected 95% confidence intervals. Unless noted otherwise, B is an unstandardized coefficient in the analysis-file metric. Composite scores on the original 1–5 scales were rescaled to M = 3.00 and SD = 0.55 before estimation; age and log-income were z-scored, and gender and migration exposure were untransformed. Coefficients for the interaction terms (a₃, b₃) are the change in the corresponding path per 1 SD of the moderator (RSS or SC). Untransformed item-level descriptives were not retained. IEDF and the mediators were mean-centred; age and log-income were z-scored; and the coefficients for interaction terms (a₃, b₃) are the change in the corresponding path per 1 SD of the moderator (RSS or SC). Because this system uses observed composite scores and treats the 420 respondents as independent observations, it does not model measurement error or the clustering of respondents within four villages; these limitations are discussed below. Measurement invariance across ethnic groups, villages, and languages was not tested.

*Qualitative phase*: Semi-structured interviews (60–90 min) were conducted with 32 residents and 8 key informants. Analysis followed reflexive thematic analysis (Braun and Clarke, 2006, 2019) in NVivo. Two coders coded the transcripts independently; Cohen’s κ (target ≥ 0.75) was used as a descriptive check on the consistency with which themes were applied, and disagreements were resolved by discussion, because reflexive approaches do not treat agreement as evidence of coding accuracy. Member-checking was used. Integration followed a joint-display approach (Fetters et al., 2013), comparing quantitative estimates with emergent themes for convergence, partial convergence, and divergence. The display was prepared after the quantitative results were final and is therefore primarily explanatory.

## 4 Results

### 4.1 Quantitative findings

Descriptive statistics for the seven scaled constructs are reported in Table 1. Place attachment (*r* = 0.46), perceived restorativeness (*r* = 0.36), and cultural identity continuity (*r* = 0.46) were positively and moderately correlated with IEDF and were also positively correlated with psychological well-being (PWB; *r* = 0.32–0.36; Table 2). RSS was moderately correlated with IEDF (*r* = 0.48) and weakly to moderately correlated with the mediators and the outcome (*r* = 0.20–0.27), whereas social cohesion (SC) was essentially uncorrelated with the other study variables (|r| ≤ 0.04) (see Figure 2).

**Table 1:** Descriptive statistics (*N* = 420).

Variable	M	SD	Min	Max
IEDF	3.00	0.55	1.17	4.83
RSS	3.00	0.55	1.24	4.81
SC	3.00	0.55	1.44	4.47
PA	3.00	0.55	1.65	4.45
PR	3.00	0.55	1.42	4.43
CIC	3.00	0.55	1.71	4.86
PWB	3.00	0.55	1.33	4.77
Age (years)	44.30	13.65	18	88
Years resident	34.96	14.51	5	80
Annual income (RMB)	15,428	8,530	2,947	61,660

M, mean; SD, standard deviation. The seven scaled constructs are reported in the analysis-file metric after each composite was rescaled to M = 3.00 and SD = 0.55; untransformed item-level descriptives were not retained. Age, years resident, and annual income are untransformed. IEDF, Indigenous Environmental Design Fidelity; RSS, ritual-spatial synchrony; SC, social cohesion; PA, place attachment; PR, perceived restorativeness; CIC, cultural identity continuity; PWB, psychological well-being.

**Table 2:** Bivariate correlation matrix.

Variable	IEDF	RSS	SC	PA	PR	CIC	PWB
IEDF	1.00						
RSS	0.48	1.00					
SC	0.04	−0.03	1.00				
PA	0.46	0.27	0.02	1.00			
PR	0.36	0.21	0.04	0.20	1.00		
CIC	0.46	0.22	−0.00	0.24	0.15	1.00	
PWB	0.34	0.20	0.03	0.32	0.36	0.34	1.00

IEDF, Indigenous Environmental Design Fidelity; RSS, ritual-spatial synchrony; SC, social cohesion; PA, place attachment; PR, perceived restorativeness; CIC, cultural identity continuity; PWB, psychological well-being.

**Figure 2:**
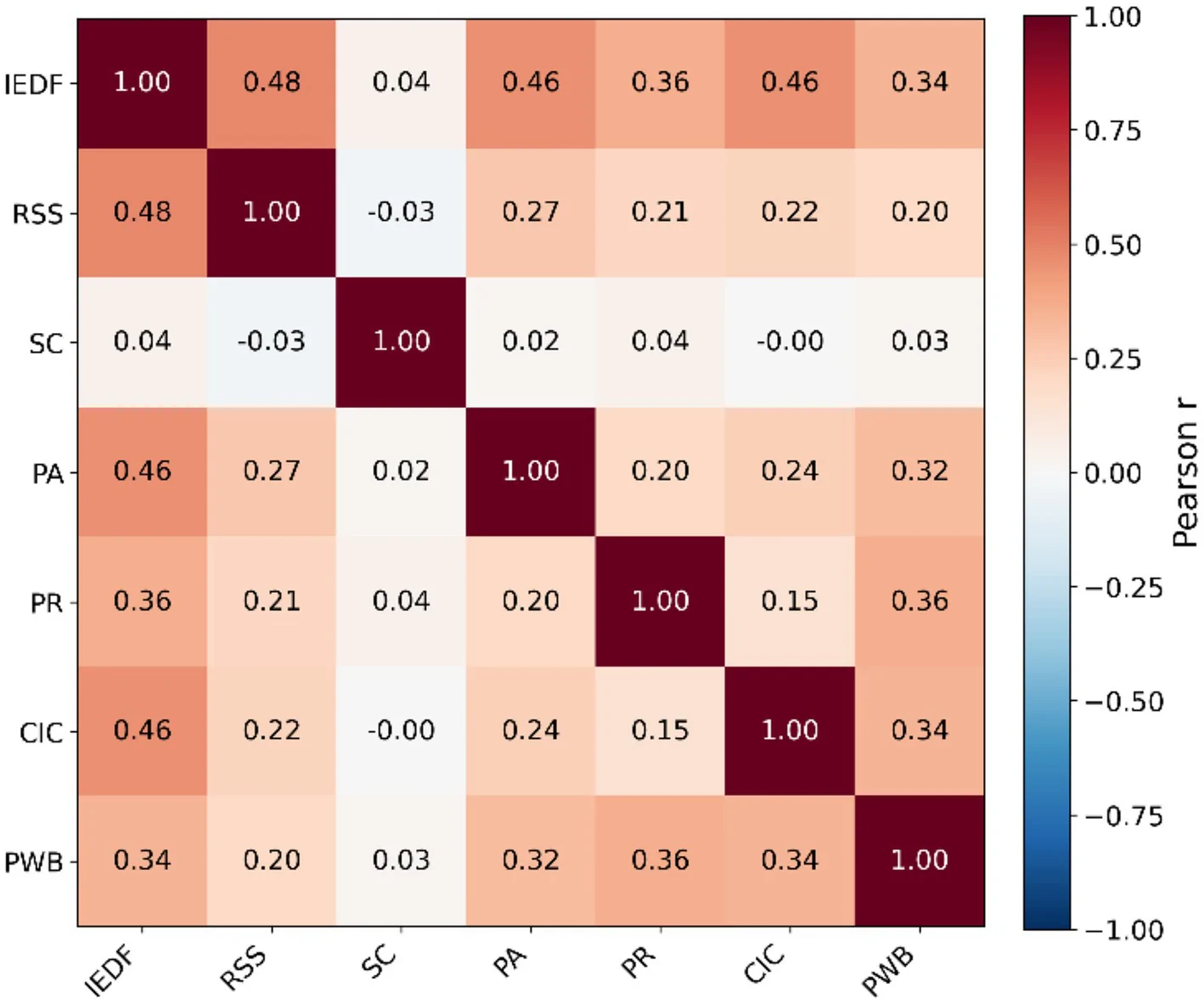
Correlation heatmap.

The seven-factor measurement model of the CL-PWB Nexus Model was assessed before hypothesis testing. Standardized loadings for all 29 items ranged from 0.68 to 0.85 (Table 3), above the 0.60 cut-off used for acceptable item loadings. In the main sample, reliability was evaluated against a single set of criteria: Cronbach’s α and composite reliability (CR) ≥ 0.70, and average variance extracted (AVE) ≥ 0.50. Cronbach’s α ranged from 0.76 (RSS) to 0.82 (IEDF, PR, CIC, and PWB), CR from 0.86 to 0.88, and AVE from 0.52 to 0.67 (Table 4), so all constructs met these criteria. The 0.80 criterion set for the pilot study was a stricter screening target for scale refinement; the RSS (α = 0.76) and SC (α = 0.77) scales fell below it in the main sample, which we note as a limitation. Discriminant validity was assessed with the Fornell–Larcker criterion (Fornell and Larcker, 1981): the square root of each construct’s AVE exceeded its correlations with the other constructs, the highest inter-construct correlation being r = 0.48 (Table 2). Measurement invariance across ethnic groups, villages, and languages was not tested (see Limitations) (see Figure 3).

**Table 3:** CFA measurement model: standardized factor loadings.

Construct	Item	Standardized loading
IEDF	IEDF1	0.83
IEDF	IEDF2	0.81
IEDF	IEDF3	0.85
IEDF	IEDF4	0.75
RSS	RSS1	0.83
RSS	RSS2	0.82
RSS	RSS3	0.81
SC	SC1	0.79
SC	SC2	0.80
SC	SC3	0.71
SC	SC4	0.79
PA	PA1	0.82
PA	PA2	0.75
PA	PA3	0.81
PA	PA4	0.77
PR	PR1	0.80
PR	PR2	0.83
PR	PR3	0.78
PR	PR4	0.81
CIC	CIC1	0.82
CIC	CIC2	0.80
CIC	CIC3	0.79
CIC	CIC4	0.79
PWB	PWB1	0.71
PWB	PWB2	0.75
PWB	PWB3	0.76
PWB	PWB4	0.68
PWB	PWB5	0.70
PWB	PWB6	0.72

Standardized loadings from the seven-factor confirmatory factor analysis (MLR); all loadings significant at *p* < 0.001.

**Table 4:** Reliability and convergent validity.

Construct	Cronbach’s α	Composite Reliability (CR)	AVE
IEDF	0.82	0.88	0.65
RSS	0.76	0.86	0.67
SC	0.77	0.86	0.60
PA	0.80	0.87	0.62
PR	0.82	0.88	0.65
CIC	0.81	0.88	0.64
PWB	0.82	0.87	0.52

**Figure 3:**
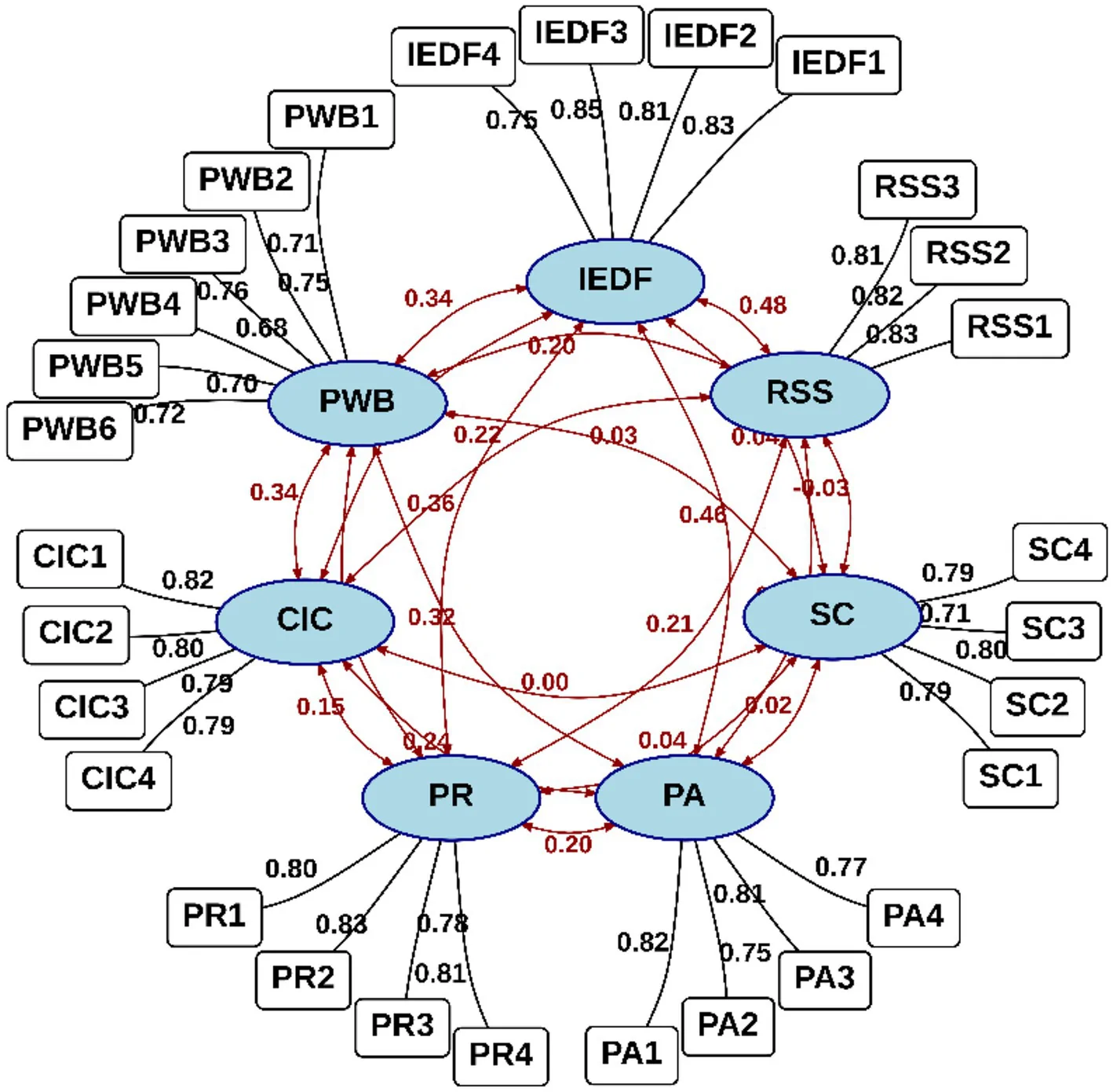
CFA measurement model.

The association of IEDF with PWB was estimated first, combined with age, gender, log-income, and migration exposure. As shown in Table 5, IEDF was significantly and positively associated with PWB (B = 0.332, SE = 0.046, t = 7.17, *p* < 0.001), supporting Hypothesis 1 and explaining 11.7% of the variance in the outcome (R^2^ = 0.117, *F*(5,414) = 11.01, *p* < 0.001). None of the covariates was significantly associated with PWB; this does not rule out confounding by other factors, such as health, education, household structure, tourism exposure, or village resources, that were not measured or modelled.

**Table 5:** Total effect model (Path c): PWB ~ IEDF + covariates.

Predictor	B	SE	*t*	*p*
IEDF	0.332	0.046	7.17	<0.001
Age (z)	0.022	0.025	0.86	0.393
Gender (male = 1)	−0.029	0.051	−0.56	0.574
Income, log (z)	0.002	0.026	0.07	0.944
Migration exposure	−0.058	0.053	−1.09	0.275

R^2^ = 0.117, Adj. R^2^ = 0.107, F(5,414) = 11.01, *p* < 0.001.

First-stage (a-path) moderated regressions (Table 6) examined whether the association between IEDF and each mediator differed by level of ritual-spatial synchrony (RSS). IEDF was significantly and positively associated with place attachment (a₁ = 0.408, *p* < 0.001), perceived restorativeness (a₁ = 0.337, *p* < 0.001), and cultural identity continuity (a₁ = 0.448, *p* < 0.001), supporting H2a–H2c. The IEDF × RSS interaction was significant for each mediator (PA, a₃ = 0.133, *p* < 0.001; PR, a₃ = 0.124, *p* = 0.002; CIC, a₃ = 0.122, *p* = 0.001), indicating that the association of IEDF with each mediator was stronger where ceremonies and daily practices were still carried out at their historically designated places (higher RSS).

**Table 6:** First-stage (a-path) moderated regressions: mediator ~ IEDF × RSS + covariates.

Mediator	IEDF (a₁)	RSS (a₂)	IEDF × RSS (a₃)	R^2^
PA	0.408***	0.037	0.133***	0.254
PR	0.337***	0.019	0.124**	0.155
CIC	0.448***	−0.002	0.122**	0.230

**p < 0.01, ***p < 0.001. Models control for age, gender, log-income, and migration exposure.

The outcome (b-path) model (Table 7) regressing PWB on IEDF, all three mediators, social cohesion, and the interaction terms between the mediators and social cohesion at the same time accounted for 28.8% of variance in well-being (R^2^ = 0.288, adjusted R^2^ = 0.267, *F*(12,407) = 13.74, *p* < 0.001). All three mediators retained significant direct associations with PWB (PA: b₁ = 0.200, *p* < 0.001; PR: b₂ = 0.272, *p* < 0.001; CIC: b₃ = 0.236, *p* < 0.001), supporting H4a–H4c. The direct association of IEDF with PWB (c′ = 0.058, *p* = 0.285) was not significant and was smaller than the total association (c = 0.332), a pattern consistent with full statistical mediation; because the design is cross-sectional, this does not establish temporal or causal mediation. The second-stage interaction terms were significant for place attachment × SC (b = 0.097, *p* = 0.030) and perceived restorativeness × SC (b = 0.106, *p* = 0.012) and marginally so for cultural identity continuity × SC (b = 0.075, *p* = 0.095), thus providing some but not consistent support for H5.

**Table 7:** Outcome (b-path) moderated regression: PWB ~ IEDF + Mediators × SC + covariates.

Predictor	B	SE	t	*p*
IEDF (c′)	0.058	0.054	1.07	0.285
PA (b₁)	0.200	0.048	4.19	<0.001
PR (b₂)	0.272	0.045	6.03	<0.001
CIC (b₃)	0.236	0.047	5.00	<0.001
SC	0.008	0.023	0.35	0.725
PA × SC	0.097	0.044	2.18	0.030
PR × SC	0.106	0.042	2.53	0.012
CIC × SC	0.075	0.045	1.67	0.095

*R*^2^ = 0.288, Adj. R^2^ = 0.267, F(12,407) = 13.74, *p* < 0.001. Model also controls for age, gender, log-income, and migration exposure (not shown).

The bootstrapped indirect associations (0.082, 95% CI [0.041, 0.127] through place attachment; 0.092, 95% CI [0.055, 0.136] through perceived restorativeness; and 0.106, 95% CI [0.060, 0.150] through cultural identity continuity) all excluded zero, supporting H6 (Table 8). For first-stage moderation by RSS, the index of moderated mediation was significant for all three mediators. For second-stage moderation by SC, the index was significant for perceived restorativeness (a₁·b₃ = 0.036, 95% CI [0.009, 0.069]) but not for cultural identity continuity (a₁·b₃ = 0.033, 95% CI [−0.014, 0.078]); for place attachment the lower confidence limit was 0.000 at three decimals (a₁·b₃ = 0.040, 95% CI [0.000, 0.085]), so the evidence for second-stage moderation is borderline (re-running the bootstrap on the analysis file with 5,000 resamples gave a lower limit of 0.0005 for the percentile interval but −0.0007 for the bias-corrected interval, so whether it excludes zero depends on the interval type). H7 was therefore partially supported.

**Table 8:** Bootstrapped (5,000 resamples) indirect effects and index of moderated mediation.

Mediator	Effect	Estimate	95% Bootstrap CI	Result
PA	Indirect (a₁·b₁)	0.082	[0.041, 0.127]	Sig.
PA	Index, Stage 1 (a₃·b₁)	0.027	[0.011, 0.045]	Sig.
PA	Index, Stage 2 (a₁·b₃)	0.040	[0.000, 0.085]	Borderline
PR	Indirect (a₁·b₁)	0.092	[0.055, 0.136]	Sig.
PR	Index, Stage 1 (a₃·b₁)	0.034	[0.012, 0.061]	Sig.
PR	Index, Stage 2 (a₁·b₃)	0.036	[0.009, 0.069]	Sig.
CIC	Indirect (a₁·b₁)	0.106	[0.060, 0.150]	Sig.
CIC	Index, Stage 1 (a₃·b₁)	0.029	[0.008, 0.052]	Sig.
CIC	Index, Stage 2 (a₁·b₃)	0.033	[−0.014, 0.078]	n.s.

CI = bias-corrected bootstrap confidence interval (5,000 resamples). Index, Stage 1 = a₃·b₁ (moderation of the IEDF–mediator path by RSS); Index, Stage 2 = a₁·b₃ (moderation of the mediator–PWB path by SC). The lower limit of the PA Stage 2 interval is 0.000 at three decimals, so this result is borderline. PA, place attachment; PR, perceived restorativeness; CIC, cultural identity continuity.

Conditional indirect associations were generally larger at higher levels of RSS and SC (Table 9). For place attachment, the conditional indirect association rose from 0.028 (95% CI [−0.008, 0.074]; not distinguishable from zero) at low RSS/SC to 0.161 (95% CI [0.087, 0.240]) at high RSS/SC, an approximately six-fold difference. For perceived restorativeness it rose from 0.035 (95% CI [0.007, 0.069]) to 0.174 (95% CI [0.106, 0.256]), approximately five-fold, and for cultural identity continuity from 0.053 (95% CI [0.011, 0.099]) to 0.177 (95% CI [0.079, 0.273]), approximately three-fold. The conditional indirect associations through perceived restorativeness and cultural identity continuity therefore remained significant, although smaller, at low RSS/SC; only the place-attachment pathway was non-significant. Because RSS and SC were set to the same level in Table 9, these contrasts cannot separate the contributions of the two moderators. When RSS and SC were varied separately (Table 9), the place-attachment pathway was non-significant at low SC at every level of RSS (for example, 0.056, 95% CI [−0.015, 0.135] at +1 SD RSS) but significant at mean SC even when RSS was low (0.055, 95% CI [0.026, 0.098]); the pathways through perceived restorativeness and cultural identity continuity were significant in all nine combinations (see Table 10). The non-significant place-attachment result at ‘low RSS/SC’ therefore reflects low social cohesion and not low ritual–spatial synchrony alone. The results of the hypothesis tests are summarised in Table 11.

**Table 9:** Conditional indirect effects of IEDF on PWB across RSS/SC levels.

Mediator	Condition	Conditional Indirect Effect	95% Bootstrap CI	Result
PA	Low (−1SD RSS/SC)	0.028	[−0.008, 0.074]	n.s.
PA	Mean (RSS = SC = 0)	0.082	[0.041, 0.127]	Sig.
PA	High (+1SD RSS/SC)	0.161	[0.087, 0.240]	Sig.
PR	Low (−1SD RSS/SC)	0.035	[0.007, 0.069]	Sig.
PR	Mean (RSS = SC = 0)	0.092	[0.054, 0.133]	Sig.
PR	High (+1SD RSS/SC)	0.174	[0.106, 0.256]	Sig.
CIC	Low (−1SD RSS/SC)	0.053	[0.011, 0.099]	Sig.
CIC	Mean (RSS = SC = 0)	0.106	[0.060, 0.154]	Sig.
CIC	High (+1SD RSS/SC)	0.177	[0.079, 0.273]	Sig.

RSS and SC were standardized and were set to the same level in each condition (Low = −1 SD, Mean = 0, High = +1 SD), so the contrasts cannot separate the contributions of the two moderators. CI = bias-corrected bootstrap confidence interval (5,000 resamples); n.s. = not significant (CI includes zero).

**Table 10:** Structural model summary (multi-equation system).

Equation	R^2^	Adjusted R^2^
Total effect (PWB ~ IEDF + covariates)	0.117	0.107
a-path: PA ~ IEDF × RSS + covariates	0.254	0.241
a-path: PR ~ IEDF × RSS + covariates	0.155	0.140
a-path: CIC ~ IEDF × RSS + covariates	0.230	0.217
b-path: PWB ~ IEDF + mediators × SC + covariates	0.288	0.267

**Table 11:** Hypothesis testing summary.

Hyp.	Test	Result	Supported
H1	Total effect regression (Table 5)	B = 0.332, *p* < 0.001	Yes
H2a	a-path regression (Table 6)	a₁ = 0.408, *p* < 0.001	Yes
H2b	a-path regression (Table 6)	a₁ = 0.337, *p* < 0.001	Yes
H2c	a-path regression (Table 6)	a₁ = 0.448, *p* < 0.001	Yes
H3	IEDF × RSS interaction (Table 6)	a₃ sig. For PA, PR, CIC	Yes
H4a	b-path regression (Table 7)	b₁ = 0.200, *p* < 0.001	Yes
H4b	b-path regression (Table 7)	b₂ = 0.272, *p* < 0.001	Yes
H4c	b-path regression (Table 7)	b₃ = 0.236, *p* < 0.001	Yes
H5	Mediator × SC interaction (Table 7)	Sig. for PA, PR; marginal CIC	Partial
H6	Bootstrapped indirect effects (Table 8)	All 95% CIs exclude 0	Yes
H7	Index of moderated mediation (Tables 8, 9)	Stage 1 significant for all three mediators; Stage 2 significant for PR, borderline for PA, not significant for CIC	Partial

*Sensitivity to village clustering*: Village-level differences were small for most constructs but substantial for IEDF (ICC(1) = 0.20, compared with 0.05 for PWB and perceived restorativeness, 0.04 for SC, 0.03 for place attachment and cultural identity continuity, and 0.01 for RSS). Village means of IEDF ranged from 2.68 (Village C) to 3.25 (Village A). Within each village the correlation between IEDF and PWB was positive (*r* = 0.15 to 0.35). Re-estimating the models with village fixed effects left the pattern of results unchanged: the total association of IEDF with PWB was B = 0.297 (SE = 0.051, *p* < 0.001); the direct association remained non-significant (c′ = 0.025, *p* = 0.655); the IEDF × RSS interaction remained significant for all three mediators (a₃ = 0.132, 0.127, and 0.121 for PA, PR, and CIC; *p* = 0.0004, 0.001, and 0.002); and the mediator × SC interactions were b₃ = 0.096 (*p* = 0.032) for PA, 0.102 (*p* = 0.015) for PR, and 0.078 (*p* = 0.083) for CIC. Standard errors clustered on village (four clusters, so these should be treated with caution) gave the same conclusions (PA × SC, *p* = 0.008; PR × SC, *p* = 0.002; CIC × SC, *p* = 0.134). Because only four villages are available, these checks cannot separate village-level from individual-level processes, and they do not resolve the confounding of village with ethnic group, tourism exposure, and degree of preservation (see Figures 4–6).

**Figure 4:**
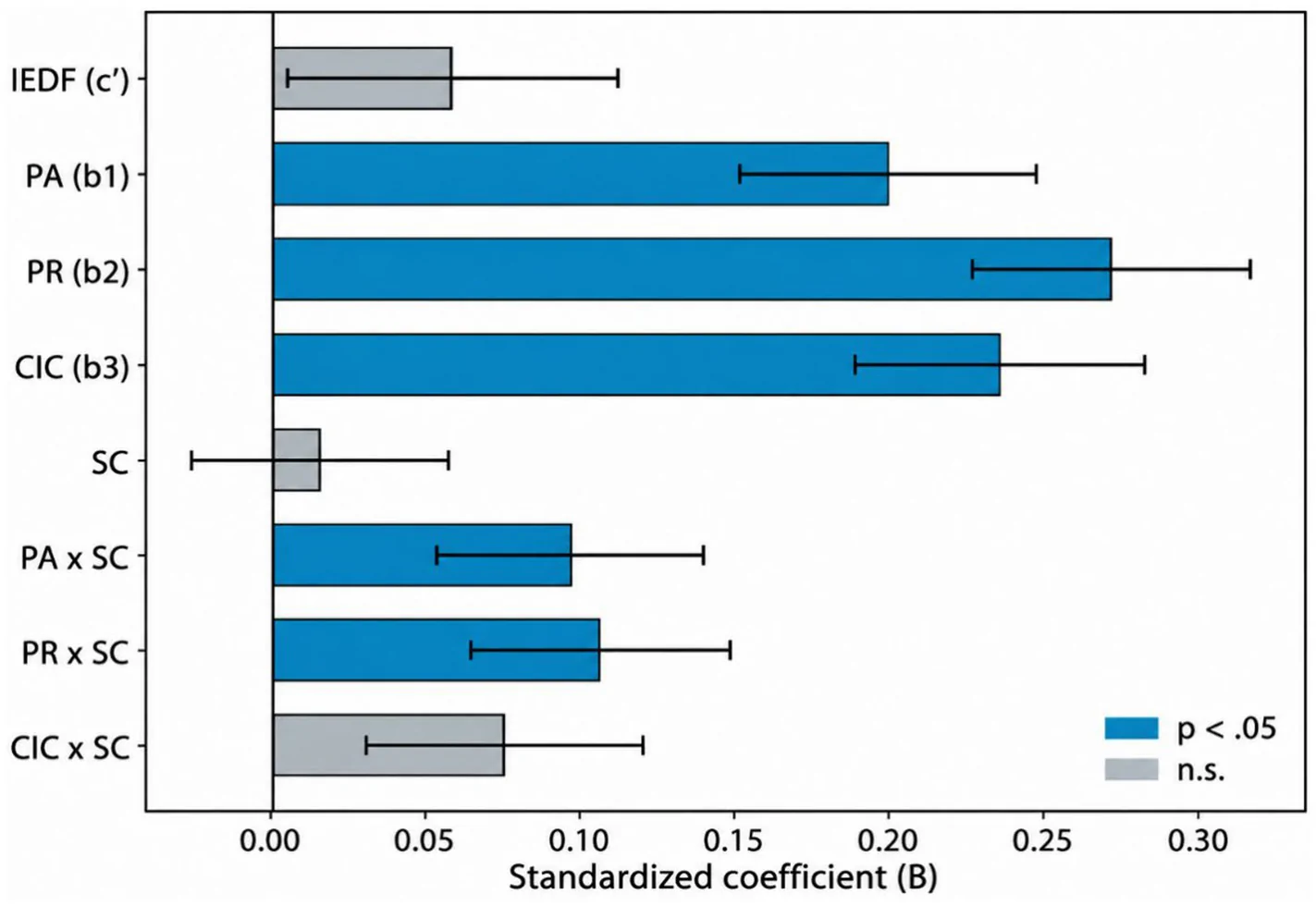
Final model coefficients.

**Figure 5:**
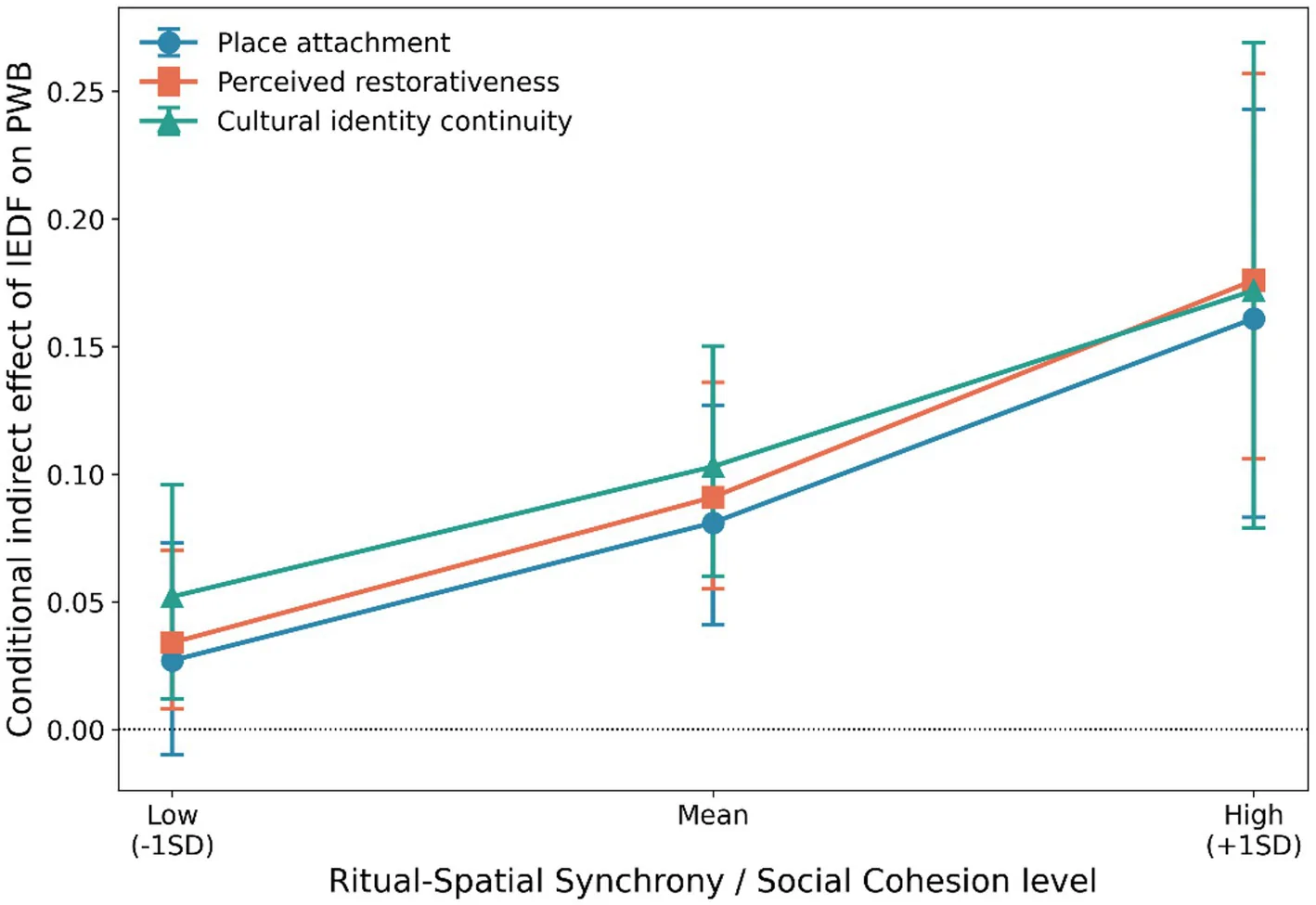
Conditional indirect associations of IEDF with PWB at low (−1 SD), mean, and high (+1 SD) levels of RSS and SC, set to the same level (95% bootstrap CIs).

**Figure 6:**
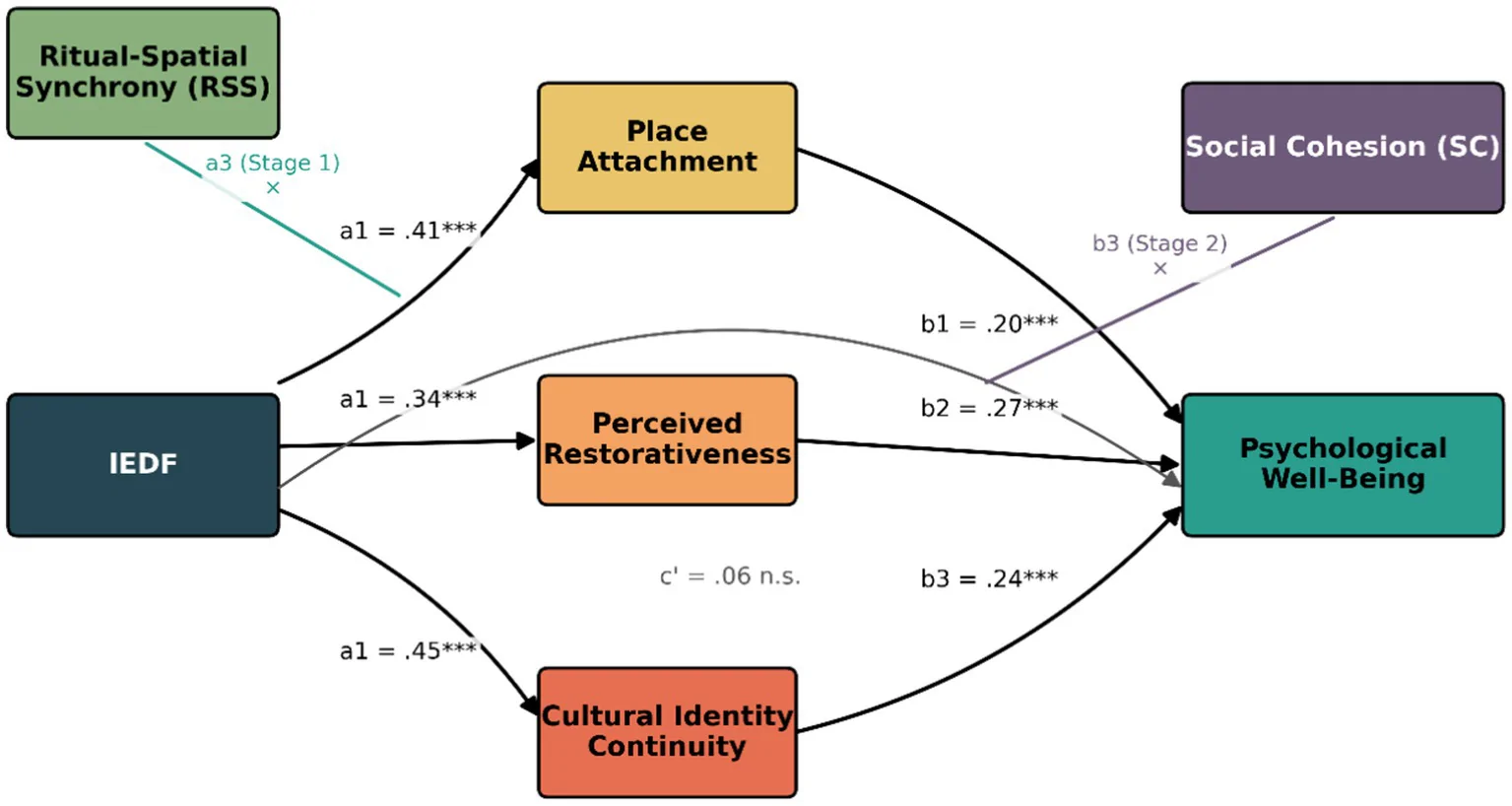
Pathway diagram.

### 4.2 Qualitative findings: thematic analysis

The 32 in-depth interviews and eight key-informant interviews were analysed thematically using a reflexive approach (verbatim transcripts, NVivo, dual coding; Cohen’s κ = 0.78–0.86 across themes), which produced four themes (Table 12). The themes were developed from the interview data and were then compared with the quantitative results in the joint display (Table 13). The correspondence between the two strands should be read in light of two facts: the interview sample was partly selected using survey scores (high versus low place attachment), and the joint display was prepared after the quantitative results were final.

**Table 12:** Themes, initial codes, frequency, and inter-rater reliability.

Theme	Initial codes	Frequency (n refs.)	Cohen’s κ
1. Landscape as ancestral memory and identity	Lineage marker, ancestor presence, felt belonging, intergenerational witness	61	0.84
2. Restorative function of traditional spaces	Mental quiet, refuge from labour, intentional calming design, sensory relief	54	0.86
3. Erosion of ritual-spatial practice	Displaced ritual site, “meaning homeless,” modern replacement structures, felt disconnection	47	0.79
4. Communal cohesion anchored in shared landscape	Shared gathering, mutual reinforcement of place and people, weakening collective use	43	0.78

**Table 13:** Joint display matrix: integration of quantitative and qualitative findings.

Quantitative result	Qualitative theme	Illustrative quote	Convergence
CIC → PWB significant (b₃ = 0.236, *p* < 0.001; Table 7)	Theme 1	“I do not do the rituals as my mother did, but the drum tower still stands, and that’s enough to make me feel like I’m still a part of something.”	Convergent
PR → PWB: largest b-path (b₂ = 0.272; Table 7); conditional indirect associations similar across pathways at high RSS/SC (0.161–0.177; Table 9)	Theme 2	“After working hard on the farm, I sit by the old pond, where my mind is still.”	Convergent
Conditional indirect associations smaller at low RSS/SC; non-significant for PA only (Table 9)	Theme 3	“Incense is still burnt during the new year, but not at the site of the old shrine; it’s in the concrete square. It does not feel the same in my chest.”	Partial convergence (smaller, not absent, at low RSS/SC for PR and CIC)
SC × mediator interactions significant for PA, PR (Table 7)	Theme 4	“the building supports the people and the people support the building.”	Convergent
SC × CIC interaction only marginal (b = 0.075, *p* = 0.095; Table 7)	Theme 4 (partial)	“The old bridge remains, but is not used as a meeting place anymore. It’s simply a piece of wood.”	Partial divergence

#### 4.2.1 Theme 1: landscape as ancestral memory and identity

There were three distinguishable sub-patterns to this theme, moving beyond a general feeling of family belonging. First, participants identified the landscape as a witness to the family history, so it was a non-volitional, but ongoing, presence that survived the participants’ memory. Second, several described an active landscape as one that teaches and communicates values through repeated physical encounter, instead of explicit instruction. Here is what a 58-year-old woman from Village A had to say: “I didn’t have anyone sit me down and teach me who we are; we learned it by sweeping the courtyard my mother swept, by bowing, where my grandmother bowed.” Third and most striking among younger participants was a continuity of identity not based on practice but proximity: a felt continuity that carried on even when the participant ceased to perform the rituals. One of the residents said, “I’m 27 years old. I do not do the rituals as my mother did, but the drum tower still stands, and that’s enough to make me feel like I’m still a part of something.” This pattern is consistent with the quantitative results: the indirect association through cultural identity continuity remained significant, and was the largest of the three, at low RSS/SC (Table 9), and its second-stage interaction with social cohesion was the weakest (Table 7), which suggests, tentatively, that identity-as-witness may depend less on active communal reinforcement than the other two mediators. A heritage officer key informant expressed a view shared indirectly by many other respondents.

#### 4.2.2 Theme 2: restorative function of traditional spaces

Participants identified at least three attributes of restoration that have been associated with design attributes. The first was sensory calm, which was due to the sound of water, shade and airflow. This was literally what one woman in Village D said: “After working hard on the farm, I sit by the old pond, where my mind is still.” The second was cognitive dissociation of labour-role identity; several participants talked about these spaces as being the only ones in which they were not “someone’s mother” or “someone’s worker.” One of the men in his 40s from Village D stated: “In the fields I always do something for someone, and at the pavilion, I am just sitting; nobody asks anything of me there.” The third, provided primarily by elders and a carpenter key informant, was designed as intentional therapy, which was an explicit statement that ancestors designed the restorative experience with the site and materials, and not just for looks: “This is no coincidence; our ancestors built the resting pavilion where the wind blows, and the water gently makes its sound, on purpose, to calm people.” This framing is consistent with the restorativeness items in the measurement model (Table 3) and offers one possible interpretation of why perceived restorativeness had the largest b-path coefficient (Table 7) while at high RSS/SC the conditional indirect associations of the three pathways were similar (0.161–0.177; Table 9). Participants emphasised physical features of the space, such as water, shade, and airflow, which may explain why restorativeness was attributed to the site itself rather than to ritual practice; however, RSS also moderated the IEDF–restorativeness path (Table 6), so this interpretation is tentative.

#### 4.2.3 Theme 3: Erosion of ritual-spatial practice under modernization

This theme had the most emotional terminology in the corpus, with two sub-patterns: substitution (ritual performed in a new, unrelated place) and abandonment (ritual discontinued once its location changed). Substitution was not reported as a loss, but rather ambivalently. One of the villagers from Village C said: “Incense is still burnt during the new year, but not at the site of the old shrine; it’s in the concrete square. It does not feel the same in my chest.” This is in line with the smaller, but for perceived restorativeness and cultural identity continuity still significant, conditional indirect associations found at low RSS/SC (Table 9). Abandonment, on the other hand, was experienced in a more clear-cut way of grief. One resident of Village C explained that planting used to be accompanied by songs to the rice spirits, but that after the terraces were levelled for the road there were no more songs. The psychological price of this displacement was narrated by the most direct voice – an elder ritualist: “Where there is a broken place, there is no meaning. You can say the words and say them, but the words have no place.” Notably, in both sub-patterns and beyond, several participants blamed this erosion not on individual choice, but on external decisions made about infrastructure and tourism development, which emerged as a clear-cut code (“externally imposed disruption”) cutting across both sub-patterns and indicating a structural, rather than purely generational, driver of ritual-spatial decline.

#### 4.2.4 Theme 4: communal cohesion anchored in shared landscape

This theme differentiated landscape-as-convener, that is, the physical site that still draws people into a collective presence, from landscape-as-relic, which is left behind but continues to exist. Measured social cohesion and design fidelity were both relatively high in Village A, and this was the most conducive setting for the convener sub-pattern. The younger informant said: “It’s not just beautiful, it’s that people continue to meet there; the building supports the people and the people support the building.” A senior member of the community in the same village went on to say: “Even our arguments take place at the drum tower — that remains together.” The relic sub-pattern was found in lower cohesion villages, and their areas were spaces that were physically “whole” but socially “empty.” One younger participant said: “The old bridge remains, but is not used as a meeting place anymore. It’s simply a piece of wood.” This difference provides a plausible qualitative explanation for the different results obtained for place attachment and restorativeness and cultural identity continuity (Table 7): communal use seems to contribute to the felt benefits of attachment and restoration in the present, whereas the continuity of cultural identity (more related to historical witnessing; Theme 1) seems to erode more slowly as the sense of communal use of a site diminishes.

The pathways in the qualitative data and in the statistical analyses (Tables 5–9) corresponded closely across the four themes, and the interviews added depth of explanation that regression coefficients cannot provide. The joint display was, however, constructed after the quantitative results were final, and the interview sample was partly selected on survey scores, so the correspondence may overstate the independence of the two strands. The clearest divergence is that cultural identity continuity was less sensitive to social cohesion, which is discussed below.

## 5 Discussion

The purpose of this study was to create and test a new conceptual model connecting indigenous environmental design to psychological well-being; to explore the concept of ritual-spatial synchrony and social cohesion as boundary conditions on the relationship between indigenous environmental design and psychological well-being; and to synthesize quantitative and qualitative evidence into a coherent and policy-relevant account. The results address these goals, but they are subject to important constraints, described below under Limitations.

As for the first goal, IEDF was positively associated with psychological well-being (H1), and this association was statistically accounted for by place attachment, perceived restorativeness, and cultural identity continuity: the direct association was not significant once the three mediators were entered, a pattern of full statistical mediation. This pattern is compatible with, but does not establish, a mechanism of the kind proposed in the model, because the data are cross-sectional and self-reported. The findings extend work on single mechanisms, such as place attachment as a mediator among older adults in Taiwan (Chen et al., 2024) and as a buffer against discrimination-related distress among Chinese migrant children (Yang et al., 2023); nature connectedness and well-being among older adults and rural low-income households in China (Zhu et al., 2025; Yang et al., 2022); cultural efficacy as a construct linking enculturation and mental health in Indigenous North American communities (Gonzalez et al., 2022); and cultural identity as a correlate of well-being among Miao, Dong, and Naxi participants in Southwest China (Zhao et al., 2026). The qualitative findings resonate with these pathways: Theme 1’s depiction of the landscape as ancestral memory resonates with cultural-continuity accounts, and Theme 2’s description of pavilions as intentionally therapeutic suggests that restorative qualities may be experienced as deliberately designed in culturally constructed settings, in contrast to the largely natural settings examined in restorativeness research (e.g., Johnson et al., 2022).

The second goal concerned the conditions under which these associations are stronger or weaker. The first-stage associations were stronger at higher RSS for all three mediators, and the second-stage association was stronger at higher SC for place attachment and perceived restorativeness (the former borderline in the index of moderated mediation) but not clearly for cultural identity continuity. Conditional indirect associations were smaller at low RSS/SC, and only the place-attachment pathway was no longer distinguishable from zero; the pathways through perceived restorativeness and cultural identity continuity remained significant. Burnette et al. (2021), studying older adults in rural and urban China, reported that social cohesion mediated the link between living arrangement and quality of life in urban but not rural areas. Our design differs (social cohesion was a moderator, in a rural-only sample, measured at the individual level), so the findings are not directly comparable, but they suggest that the role of social cohesion may vary by mechanism, being stronger for attachment and restoration than for identity continuity. The smaller conditional indirect associations at low RSS/SC, together with Theme 3’s accounts of ritual substitution and abandonment, are consistent with studies of landscape erosion in Hani and Yao villages (Ma et al., 2023; Gu et al., 2025) and with rural-migration studies (Deng et al., 2023; Zhou et al., 2022), in that disruption may involve not only changes to physical structures but also the displacement of practice. Varying the moderators separately showed that the place-attachment pathway was non-significant at low SC regardless of RSS, and significant at mean SC even when RSS was low (Table 9). Because RSS and SC were examined at the same level and village-level differences are confounded with these variables, these interpretations are tentative.

### 5.1 Implications

Theoretically, this study links two literatures that have rarely been connected, research on structural erosion in heritage conservation and landscape planning (Gu et al., 2025; Ma et al., 2023) and psychological research on well-being correlates (Zhao et al., 2026; Xu et al., 2023), by examining them within a single mediated–moderated model. In practice, the results are compatible with the view that heritage policy focused on physical recovery alone may be insufficient: the association of design fidelity with well-being was accounted for by the mediators, and the pathways were weaker at lower levels of ritual use and communal cohesion. Because the data are cross-sectional, they cannot show that restoring ritual use or cohesion would improve well-being; intervention-oriented and longitudinal designs are needed. These considerations are in keeping with culturally responsive Indigenous mental-health practice in Australia (Darnett et al., 2024) and with the association between cultural continuity and mental health among urban Indigenous people in Toronto (Firestone et al., 2022).

### 5.2 Contribution to theory

The constructs in the model are individually well established, and we do not claim that they are new; the contribution is a more specific account of how a landscape-level condition (design fidelity) is related to well-being. First, for place attachment theory (Hidalgo and Hernández, 2001; Lewicka, 2011; Scannell and Gifford, 2010), the findings suggest that attachment may operate as one of several parallel pathways and not as a sole explanatory link, and that its association with well-being was stronger where communal life (SC) was stronger. Second, for attention restoration theory (Kaplan, 1995; Kaplan and Kaplan, 1989), the findings are compatible with the idea that culturally constructed settings can be experienced as restorative, with the interviews suggesting that restoration is attributed to deliberate ancestral design, an aspect rarely considered in studies of natural settings. Third, for cultural continuity theory (Chandler and Lalonde, 1998), the contrast between symbolic continuity (a structure that stands) and behavioural continuity (a structure that is used) was reflected in the smaller conditional indirect associations at low RSS and, conversely, in Theme 1’s accounts of continuity through proximity even where ritual had lapsed; together these point to a possible boundary condition on the continuity-as-use argument that deserves direct testing. Overall, the contribution lies in specifying boundary conditions (ritual–spatial coupling and communal cohesion) on pathways that are usually studied separately, not in identifying new mechanisms.

### 5.3 Cultural specificity

The four villages represent different ethnic groups (Dong, Miao, Buyi, and Naxi) with distinct histories, languages, and ritual traditions, yet the same pathways were estimated for all respondents. The interviews suggest that the meanings attached to particular structures (drum towers, wind-and-rain bridges, ponds, shrines, terraces) are locally specific, and that intergenerational relationships shape how landscapes are experienced: elders described learning through repeated physical encounter, whereas younger participants described continuity through proximity even when they no longer performed rituals. Whether the constructs have equivalent meaning across ethnic and linguistic groups was not tested, and the term ‘Indigenous’ is used as a broad analytic label (see Terminology) and not as a claim that these groups share a single cultural framework (Smith, 2012). Group-specific analyses and measurement-invariance testing (Cheung and Rensvold, 2002) are priorities for future work.

### 5.4 Limitations

This study has several limitations. First, the cross-sectional design does not allow causal or temporal inference; mediation and moderation here describe statistical patterns of association, and alternative directions are plausible (for example, residents with higher well-being may perceive greater design fidelity or participate more in ritual); longitudinal or panel designs would be needed to address this (Yang et al., 2023). Unmeasured confounders such as health, education, household structure, tourism exposure, village resources, and baseline socioeconomic conditions may account for part of the associations, and the non-significance of the four demographic covariates entered does not rule out confounding. Second, all constructs were measured by self-report at a single time point, so common-method variance may inflate associations (Podsakoff et al., 2003); the discriminant validity (Henseler et al., 2015; Sagone et al., 2023, Shen et al., 2025) obtained in the CFA does not address this. Third, respondents are nested in only four purposively selected villages that differ simultaneously in ethnic-cultural context, tourism exposure, and preservation. The analyses treated the 420 respondents as independent, did not model clustering, and used an individual-level measure of social cohesion; with four clusters, village-level moderation cannot be estimated reliably (Maas and Hox, 2005; Raudenbush and Bryk, 2002), so the findings concerning SC (H5 and H7 in particular) should be regarded as exploratory (village sample sizes, intraclass correlations, and fixed-effects and cluster-robust sensitivity analyses are reported in Section 4.1; they support the same pattern of results but cannot resolve the confounding of village with ethnic group, tourism exposure, and preservation). Fourth, the IEDF index and the RSS checklist were developed for this study; evidence of their validity beyond the factor structure, reliability, and Delphi procedures reported here is limited, the RSS (α = 0.76) and SC (α = 0.77) scales fell below the pilot target of α ≥ 0.80, and measurement invariance across ethnic groups, villages, and languages was not tested. Fifth, the regression-based path analysis used observed composite scores and therefore did not model measurement error; a multilevel latent-variable model would be a stronger test. Sixth, the proximity strata were used for sampling only, and no comparison across levels of design intactness was conducted. Seventh, Table 1 reports the seven scaled constructs after rescaling to a common mean and standard deviation, and the untransformed descriptives were not retained; readers therefore cannot judge the original score distributions from this table. Eighth, the findings may not generalize beyond ethnic minority villages in Southwest China, and comparative replication is needed (Yepthomi et al., 2025; Gonzalez et al., 2022). Ninth, the joint display was constructed after the quantitative results were final and the interview sample was partly selected using survey scores, so the correspondence between strands may overstate their independence; the partial divergence for cultural identity continuity and social cohesion remains unexplained and merits further qualitative work. Lastly, as with Ma et al. (2023) and Gu et al. (2025), this study gives a snapshot of landscapes in the midst of modernization, and the associations observed here may underestimate psychological impacts of erosion that are yet to occur.

## 6 Conclusion

The results of this study suggest that, in the villages studied, the fidelity and the continued use of traditional cultural landscapes are both relevant to residents’ psychological well-being and are best considered together. Indigenous Environmental Design Fidelity was positively associated with well-being, and this association was statistically accounted for by place attachment, perceived restorativeness, and cultural identity continuity. The indirect associations were generally stronger where ritual practice remained spatially tied to the landscape and where communal cohesion was stronger, and weaker where it was not; the pathway through place attachment was no longer distinguishable from zero at low levels of both. Interview themes of ancestral memory, designed restoration, ritual displacement, and communal convening complemented these patterns and suggest that structural preservation may carry little psychological value unless the landscape is lived in. Because the design was cross-sectional and based on self-report, these findings describe associations and not causal effects. The CL-PWB Nexus Model offers a framework for further longitudinal and multilevel testing, and a cautionary note for heritage and rural-development policy: protecting the form of Indigenous environmental design while allowing ritual practice and use of the landscape to erode may preserve the form but not the conditions under which it is associated with well-being.

## Data Availability

The original contributions presented in the study are included in the article/supplementary material, further inquiries can be directed to the corresponding author.
